# Clonal Relationships Impact Neuronal Tuning within a Phylogenetically Ancient Vertebrate Brain Structure

**DOI:** 10.1016/j.cub.2014.07.015

**Published:** 2014-08-18

**Authors:** Alistair M. Muldal, Timothy P. Lillicrap, Blake A. Richards, Colin J. Akerman

**Affiliations:** 1 Department of Pharmacology, University of Oxford, Oxford OX1 3QT, UK; 2 Department of Cell and Systems Biology, University of Toronto, Toronto, ON M5S 3G5, Canada

## Abstract

Understanding how neurons acquire specific response properties is a major goal in neuroscience. Recent studies in mouse neocortex have shown that “sister neurons” derived from the same cortical progenitor cell have a greater probability of forming synaptic connections with one another [1, 2] and are biased to respond to similar sensory stimuli [3, 4]. However, it is unknown whether such lineage-based rules contribute to functional circuit organization across different species and brain regions [5]. To address this question, we examined the influence of lineage on the response properties of neurons within the optic tectum, a visual brain area found in all vertebrates [6]. Tectal neurons possess well-defined spatial receptive fields (RFs) whose center positions are retinotopically organized [7]. If lineage relationships do not influence the functional properties of tectal neurons, one prediction is that the RF positions of sister neurons should be no more (or less) similar to one another than those of neighboring control neurons. To test this prediction, we developed a protocol to unambiguously identify the daughter neurons derived from single tectal progenitor cells in *Xenopus laevis* tadpoles. We combined this approach with in vivo two-photon calcium imaging in order to characterize the RF properties of tectal neurons. Our data reveal that the RF centers of sister neurons are significantly more similar than would be expected by chance. Ontogenetic relationships therefore influence the fine-scale topography of the retinotectal map, indicating that lineage relationships may represent a general and evolutionarily conserved principle that contributes to the organization of neural circuits.

## Highlights

• We perform in vivo fate mapping of single progenitors in *Xenopus laevis* optic tectum
• Two-photon calcium imaging reveals similar receptive fields in sister tectal neurons
• Clonal relationships influence the fine-scale topography of the retinotectal map
• This supports a conserved influence of lineage on neuronal response properties

## Teaser

By labeling single neuronal clones in the *Xenopus* optic tectum and mapping visual response properties using calcium imaging, Muldal et al. show that sister neurons have more similar receptive field positions than nonsisters. This provides the first evidence that clonal relationships influence neuronal function in a noncortical brain structure.

## Results and Discussion

**Figure 1:**
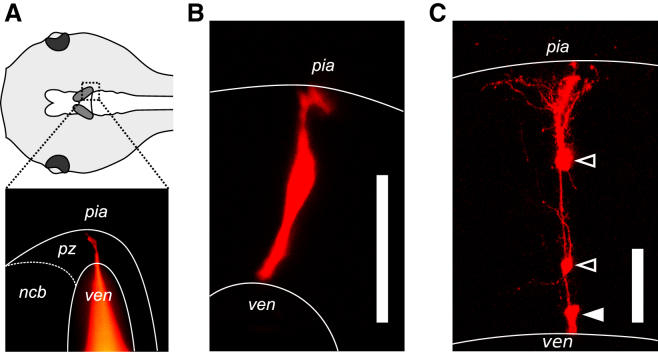
Lineage Tracing of Individual Tectal Progenitor Cells (A) Schematic dorsal view of a tadpole’s head (top) illustrating the positions of the two optic tecta (shaded); epifluorescence image (bottom) showing the electroporation of a single tectal progenitor cell with a fluorescently conjugated dextran (red). Region corresponds to dashed box above. ven, ventricle; pz, proliferative zone; ncb, neuronal cell bodies. (B) Two-photon image showing a single tectal progenitor cell captured 2 hr postelectroporation. The scale bar represents 50 μm. (C) Image of a tectal clone consisting of one radial progenitor cell (solid arrowhead) and two daughter neurons (open arrowheads), collected 10 days postelectroporation. The scale bar represents 50 μm.

To examine whether lineage-based rules contribute to functional circuit organization in the optic tectum, we developed a method for labeling a single neuronal clone per animal, which enabled us to definitively identify sister tectal neurons. All animal procedures were conducted in accordance with UK Home Office regulations. Individual tectal progenitor cells in the proliferative zone [8] of stage 44–47 *Xenopus laevis* tadpoles (7–16 days postfertilization) were targeted for single-cell electroporation with a dextran-conjugated red fluorescent dye (Figure 1A; Supplemental Experimental Procedures available online) [9]. This dye does not leak out of cells and is only passed on to daughter cells [10, 11]. To ensure that a single neuronal clone was labeled, we conducted in vivo two-photon imaging at different time points. The first imaging was conducted 1–3 hr after electroporation to be certain that only one progenitor had taken up the dextran (Figure 1B). From a total of 438 animals in which we confirmed that a single progenitor cell was labeled, 103 contained two or more labeled sister neurons when the animal was reimaged 6–19 days later at stage 49 or 50 (Figure 1C).

**Figure 2:**
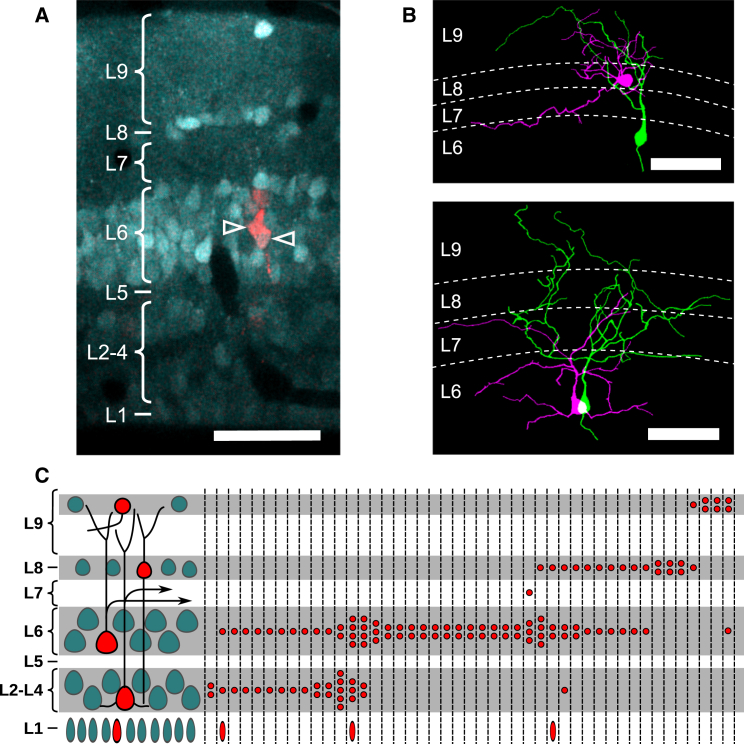
Morphology and Laminar Distribution of Tectal Sister Neurons (A) Two-photon image showing a pair of labeled sister neurons (red, open arrowheads) within a tectum loaded with OGB1-AM (cyan). The scale bar represents 50 μm. Boundaries of the nine tectal layers are annotated on the left. (B) Example morphological reconstructions of labeled sister neurons located in different tectal layers (top) and in the same tectal layer (bottom). Dotted white lines denote the positions of the layer boundaries, as determined from the OGB1-AM loading. Scale bars represent 50 μm. (C) Diagram showing the main tectal layers and cell types (left) and laminar fates of labeled sister neurons (right; n = 45 clones). Cell-dense layers are gray; neuropil layers are white. Red circles within each dashed column represent layer positions of daughter neurons generated by a single progenitor cell.

To reveal the architecture of the tectum and to probe the functional properties of tectal neurons, we then injected the calcium indicator dye Oregon Green BAPTA1-AM (OGB1-AM) into the region encompassing the dextran-labeled neurons. Images taken before and after the OGB1-AM injection enabled us to confirm 45 animals in which the dextran-labeled neurons could still be clearly distinguished, and the different tectal layers were clearly demarcated [12, 13] (Figure 2A). Each clone was comprised of 2–7 fluorescently labeled neurons. The majority of clones (25 out of 45; 56%) spanned multiple cell-dense layers of the tectum, and, in the remainder (20 out of 45; 44%), the neurons were restricted to the same layer (Figure 2B). Across all clones, there was a strong tendency for neurons derived from the same progenitor to be situated within nearby cell-dense layers (p < 2 × 10^−5^, bootstrap test; Figure 2C; Supplemental Experimental Procedures).

**Figure 3:**
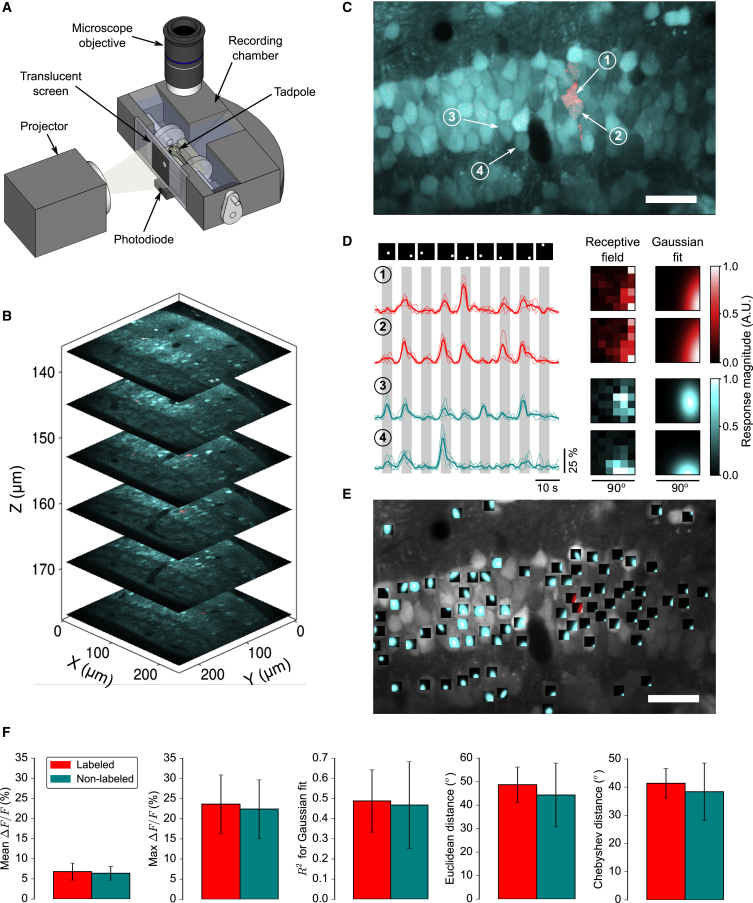
Two-Photon Calcium Imaging of Sister Neurons and Nearby Nonsister Neurons in the Optic Tectum (A) Experimental setup for in vivo calcium imaging and visual stimulation. (B) Two-photon stack through a region of tectum containing a single clone (red) and loaded with OGB1-AM (cyan). The z axis represents depth relative to the pial surface. (C) Single plane containing two dextran-labeled sister neurons. The scale bar represents 50 μm. (D) Example traces showing visually evoked calcium responses recorded from the neurons labeled in (C). Thin lines denote single trials; thick lines denote the mean response across trials. The corresponding visual stimuli are shown above. Raw spatial RFs and Gaussian fits corresponding to these neurons are also shown (right). (E) Fitted spatial RF maps obtained simultaneously from the two labeled sister neurons (red) and 83 nearby nonsister tectal neurons (cyan) shown in (C), superimposed onto their respective soma positions. The scale bar represents 50 μm. (F) Population data showing that clonally labeled tectal neurons do not differ from nonlabeled neurons in terms of their response magnitude (mean ΔF/F, p = 0.35; maximum ΔF/F, p = 0.31; n = 11 labeled neurons and n = 531 nonlabeled neurons; Mann-Whitney U test), their spatial selectivity (R^2^ values for Gaussian RF fits, p = 0.34), or the eccentricities of their RFs, as measured by either the euclidean (p = 0.16) or Chebyshev (p = 0.24) distance from the center of the stimulus area to the center of the fitted RF. Plots indicate mean ± SD.

We then used two-photon calcium imaging to assess the response properties of clonally related neurons. We mapped spatial receptive fields (RFs) by simultaneously recording visually evoked calcium responses in both dextran-labeled and nonlabeled tectal neurons in the same animals (Figures 3A–3C; Supplemental Experimental Procedures) [14, 15, 16]. For clones to be included in the analysis, labeled neurons were required to exhibit robust spatially localized RFs, as determined statistically by fitting each RF with a 2D Gaussian function (Figures 3D and 3E; Supplemental Experimental Procedures). Clones in which only one neuron satisfied these criteria had to be excluded because sister comparisons were not possible. Under these criteria, we obtained a subset of animals with significant spatially selective responses in multiple dextran-labeled sister neurons and in a large fraction of nonlabeled neighboring neurons (11 labeled neurons, 531 nonlabeled neurons, four animals). Importantly, there was no significant difference between labeled and nonlabeled neurons in terms of their response amplitudes, the quality (R^2^) of the RF fits, or the eccentricity of their RF centers (Figure 3F).

**Figure 4:**
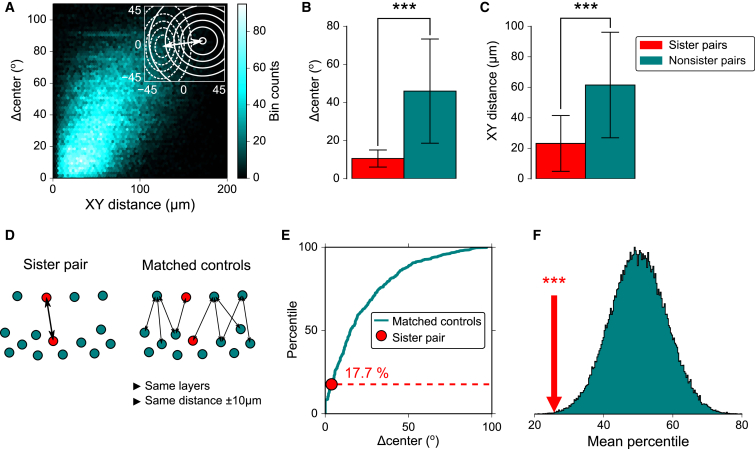
Sister Neurons in the Optic Tectum Have More Similar Spatial RFs Than Nonsisters (A) Relationship between spatial distance and Δcenter value for all pairs of tectal neurons (^∗∗∗^p < 0.001, ρ = 0.45, Spearman correlation). Inset illustrates how the Δcenter value was computed for a pair of RFs. (B) Δcenter values for sister and nonsister pairs of tectal neurons (data indicate mean ± SD; n = 13 clonal pairs and n = 72,546 nonclonal pairs from four animals in which a single clone was labeled; ^∗∗∗^p < 0.001, Mann-Whitney U test). (C) Spatial distances between somata of sister and nonsister pairs (^∗∗∗^p < 0.001, U test). (D) Schematic showing a pair of sister neurons and its corresponding set of spatially matched nonsister control pairs. (E) Cumulative distribution of Δcenter values for an example pair of sister neurons and its set of matched nonsister control pairs. (F) Pairwise bootstrap test confirms that sister neurons have more similar RF center positions than would be expected, given their spatial proximity within the tectum. Cyan distribution represents a random sample of mean percentile values (Supplemental Experimental Procedures). The mean percentile for sister pairs (red arrow) was significantly smaller than would be expected by chance (^∗∗∗^p < 0.001, bootstrap test).

These data provided the opportunity to test whether clonal relationships influence the RF properties of tectal neurons. To quantify functional differences between pairs of tectal neurons, we computed the euclidean distance between the centers of their fitted RFs (Δcenter; Figure 4A). As expected, given the retinotopic organization of the tectum, there was a significant positive correlation between the spatial separation of pairs of neurons and their Δcenter values (Figure 4A). Although pairs of sister neurons had smaller Δcenter values than nonsister pairs (Figure 4B), they also tended to be situated closer to one another within the tectum (Figure 4C). Thus, to assess the effect of clonal relationships, it was crucial for us to control for the bias introduced by this spatial clustering. We therefore compared each pair of sister neurons with a spatially matched set of nonsister control pairs (Figure 4D; Supplemental Experimental Procedures). The spatially matched control pairs had to be situated in the same combination of tectal layers, and they had to be the same distance apart as the corresponding sister pairs, to within a tolerance of ±10 μm. We expressed the degree of functional similarity between each sister pair relative to its matched controls as a percentile (Figure 4E; Supplemental Experimental Procedures). Percentile values less than the median indicate neuron pairs that had more similar RF center positions than their average matched control pair. Across the population, we found that sister pairs had a significantly smaller average percentile value than would be expected by chance (p < 0.001, bootstrap test; Figures 4F and S1A; Supplemental Experimental Procedures). Thus, pairs of sister neurons show more similar RF center positions than would be expected, given their spatial proximity within the tectum. This bias was also evident when we excluded pairs of neurons located within the same tectal layer (Figure S1B).

Our data demonstrate that sister neurons within the optic tectum have significantly more similar RF centers than nonsisters, indicating that neuronal lineage relationships influence the fine-scale topography of the retinotectal map. This is consistent with the observation that clonally related neurons can show similar orientation preferences in mouse visual cortex [3, 4]. The functional significance of such a mechanism is not yet fully understood, but it has been proposed that lineage relationships contribute to the establishment of precise canonical microcircuits [1, 5]. Given that retinotopic map formation has been shown to be controlled by molecular gradients and neuronal activity [17, 18], the influence of lineage upon a tectal neuron’s functional properties could reflect the inheritance of a particular profile of gene expression [19, 20] and/or activity-dependent processes [2, 4]. Fundamentally, the fact that clonal relationships influence responses in the optic tectum, an ancient brain structure that is common to all vertebrates, indicates that lineage relationships may represent a general and evolutionarily conserved principle that contributes to the organization of neural circuits.

## Author Contributions

A.M.M., B.A.R., and C.J.A. designed the experiments. A.M.M. and B.A.R. performed the experiments. A.M.M. and T.P.L. performed the analysis. A.M.M. and C.J.A. wrote the paper.

## Supplemental Information

Document S1. Supplemental Experimental Procedures and Figure S1

Document S2. Article plus Supplemental Information
