# Supplementary material for: Clonal Relationships Impact Neuronal Tuning within a Phylogenetically Ancient Vertebrate Brain Structure
Source: Curr Biol. 2014 Aug 18;24(16):1929–33. doi: 10.1016/j.cub.2014.07.015 (PMC4151134; doi:10.1016/j.cub.2014.07.015)
Supplement: Document S1. Supplemental Experimental Procedures and Figure S1 [file mmc1.pdf]

Current Biology, Volume *24*

Supplemental Information

Clonal Relationships Impact Neuronal Tuning within a Phylogenetically Ancient Vertebrate Brain Structure

Alistair M. Muldal, Timothy P. Lillicrap, Blake A. Richards, and Colin J. Akerman

Figure S1

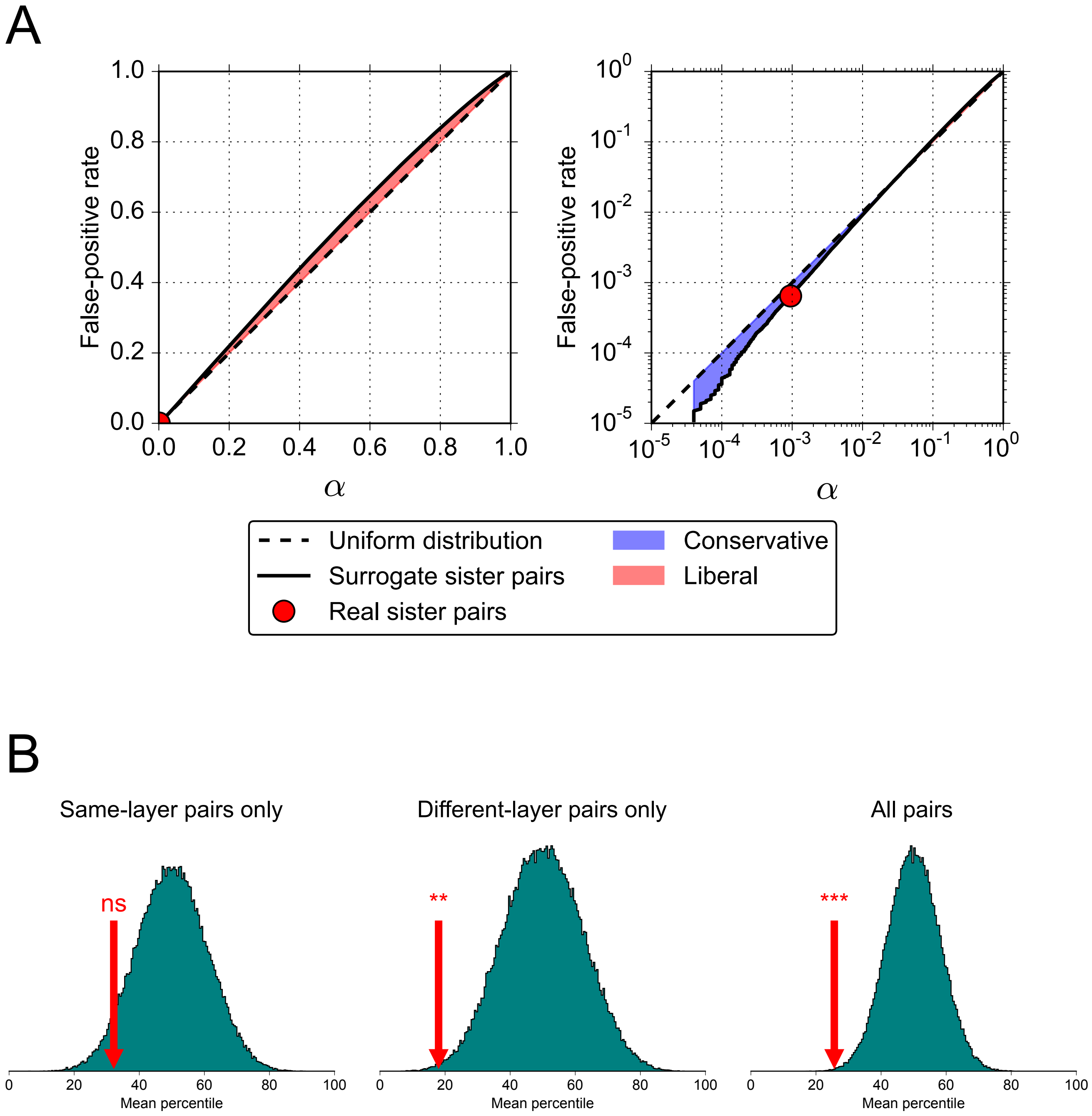

A
False-positive rate
1.0
0.8
0.6
0.4
0.2
0.0
0.0
0.2
0.4
0.6
0.8
1.0
α
10^0
10^{-1}
10^{-2}
10^{-3}
10^{-4}
10^{-5}
Uniform distribution
Surrogate sister pairs
Real sister pairs
Conservative
Liberal
B
Same-layer pairs only
Different-layer pairs only
All pairs
ns
**
***
0
20
40
60
80
100
Mean percentile

Figure S1, related to Figure 4

(A) To confirm that our pairwise bootstrap test did not exhibit a bias towards Type I errors (i.e. incorrectly rejecting the null hypothesis that sister pairs do not show smaller Δcenter values than non-sister pairs), we performed an additional statistical control to estimate the false-positive rate of the test [S1]. For each real pair of sister cells we randomly selected one of its spatially matched non-sister control pairs to serve as a surrogate. We then treated each surrogate sister pair to the same analysis we performed on the real sister pairs. By doing this for 1 million sets of surrogate pairs we generated a sample of p-values from pairs of cells that were similar to real sister pairs in terms of their relative spatial positions, but that lacked any specifically defined lineage relationship. The plots show the cumulative probability distributions for the p-values we obtained from the surrogate pairs (solid black lines), plotted on both linear (left) and log-log (right) axes. These are equivalent to plotting the estimated false-positive rate against the critical value (α) of the pairwise bootstrap test. If the test were perfectly unbiased, the p-values we obtained from the surrogate pairs would be uniformly distributed between 0 and 1, such that the false-positive rate is exactly equal to the critical value of the test (dashed lines). For example, when $\alpha = 0.05$, the probability that a perfectly unbiased test would incorrectly reject the null hypothesis is exactly 5%. If our pairwise bootstrap test is performed with $\alpha = 0.05$, the corresponding false-positive rate of 0.052 means that there is a 5.2% chance of incorrectly rejecting the null hypothesis, and therefore the test is slightly too liberal at this critical value. However, we find that p-values less than 0.0286 are actually under-represented relative to a uniform distribution (blue shaded region). Since the p-value we obtained for the real sister pairs falls within this region ($P < 0.00095$, red circle), our original p-value was too conservative. We can correct for this bias by reading off the false-positive rate at $\alpha = 0.00095$. This gives a corrected p-value of 0.00064, allowing us to safely reject the null hypothesis at the 0.1% level.

(B) To examine whether the bias for clonally related neurons to have similar RF center positions could be detected for cells either within or across tectal layers, we divided our set of 13 sister pairs into two subsets: one consisting of pairs where both neurons were within the same layer, and the other consisting of pairs where the neurons were situated in different layers. We then performed a pairwise bootstrap test separately on each subset of pairs (Supplemental Experimental Procedures). The plots show the results of these bootstrap tests, plotted according to the conventions of Fig. 4F. The result obtained for all sister pairs is also shown for reference (identical to Fig. 4F). We found that different-layer pairs showed a strong and highly statistically significant bias towards having more similar RF center positions (mean percentile = 18.0%; $P < 0.005$; n = 6 pairs). Although same-layer pairs also had a lower average percentile than their matched controls, the magnitude of the effect was smaller and did not reach statistical significance at the 5% level (mean percentile = 32.2%; $P = 0.051$; n = 7 pairs).

Supplemental Experimental Procedures

Single-cell electroporation

All animal procedures were conducted in accordance with UK home office regulations. Wild-type *Xenopus laevis* tadpoles staged between 44 and 47 according to [S2] were anesthetized in 0.01 % wt/vol tricaine methane sulphonate (MS222). A glass micropipette filled with a fluorescently-labeled dextran (Alexa Fluor 594 dextran conjugate, 10,000 MW, anionic, fixable; Molecular Probes) was inserted into the right ventricle. The tip was then advanced until it touched the ventricular wall of the proliferative zone, located at the medial-posterior boundary of the tectum [S3], and a single positive voltage pulse (+1 V amplitude, 2 ms duration) was delivered using an Axoporator 800A (Axon Instruments). Within 3 h of electroporation animals were screened under a two-photon microscope and any that showed either multiple fluorescent cells, or cells that did not morphologically resemble radial progenitor cells, were discarded.

In vivo imaging

The right tectum of each tadpole was bulk-loaded with the calcium-sensitive fluorescent indicator Oregon Green 488 BAPTA-1 AM (OGB1-AM; Molecular Probes). This was prepared at a concentration of 10 mM in DMSO containing 20 % pluronic acid (Sigma), which was further diluted 10:1 in calcium-free Ringer's solution (in mM: 150 NaCl, 2.5 KCl, 10 HEPES). One microlitre of Alexa 594 red fluorescent dye (Molecular Probes) was added to aid visualization during loading. The tadpole was anesthetized in MS222, and the dye solution was pressure-injected into the tectal parenchyma through a glass micropipette using a Picospritzer III (General Valve). The tadpole was allowed to fully recover in normal rearing solution (1 – 3 hrs) and then was immersed in rearing solution containing 0.3% wt/vol pancuronium dibromide (Sigma) for 15 min to prevent muscle movements during functional imaging. The tadpole was positioned within a custom-made recording chamber with the eye contralateral to the loaded tectum facing a translucent screen onto which visual stimuli were projected (**Fig. 3A**). All imaging was carried out using a custom-built two-photon microscope consisting of a modified confocal scan unit (Olympus FV300) and a Ti:Sapphire laser (Newport Spectra-Physics Mai Tai HP). The laser wavelength was tuned to 810 nm to simultaneously excite both the Alexa 594-conjugated dextran and the OGB1-AM calcium dye. Emission from the red and green fluorophores was separated using a dichroic mirror (Q595LP, Chroma) and corresponding bandpass filters (HQ645/75M and HQ525/50M-2P, Chroma) and detected using separate photomultplier tubes. During visual stimulation, *x-y* raster scans were captured at 2 Hz over a rectangular region of tectum measuring approximately 170x110 μm, at depths of 140-260 μm from the pial surface.

Visual stimulation

The setup for visual stimulation was similar to that previously described [S4]. Visual stimuli were generated using custom software written in Python and were projected onto the window of the imaging chamber using an LCD projector (Samsung SP-P310ME; 800 x 600 px, 60 Hz refresh rate) masked by a Wratten Filter 32 (Kodak). The tadpole was positioned such that the stimulus window covered the central 90° x 90° of the tadpole's left visual field. To ensure that the imaging data was precisely time-locked to the presentation of the visual stimuli, a photodiode positioned in front of the projector was used to trigger the acquisition of each frame. The stimuli consisted of bright dots with a radius of 12.9° which briefly appeared against a dark background. The minimum and maximum luminance values of the image projected onto the screen were 0.06 cd/m^2 and 1025 cd/m^2, respectively. During each stimulus epoch, a dot appeared at a pseudorandom location within a 6 x 6 grid for 2 s, followed by a 10 s blank period. Each stimulus position was repeated 3-5 times per

experiment.

Laminar and morphological analysis of sister neurons

Tectal layers were identified based on cell density and neuropil staining from two-photon stacks through OGB1-AM loaded tissue, in accordance with [S5]. We performed a bootstrap test to establish whether clonally-related cells tend to occupy neighboring layers of the tectum. Only layers 2-4, 6, 8 and 9, which are neuron-dense, were included in this analysis. Layers 2-4 cannot be reliably distinguished at this developmental stage and were therefore grouped together. From our experimental data set of 45 clones, we generated one million surrogate data sets in which we randomly shuffled the neurons within each layer across the clones. The real and surrogate data were then compared in terms of the average laminar distance between all possible pairs of neurons within each clone, where distance was defined as the absolute difference in the laminar positions of the neurons. We obtained a p-value by calculating the proportion of surrogate data sets that had a smaller average pairwise distance than the experimental data. In a subset of clones where the neuronal processes were sufficiently well-filled by the fluorescent dextran (n = 15/45), labeled sister neurons were reconstructed from two-photon stacks using the Simple Neurite Tracer plugin for FIJI [S6].

Processing of calcium imaging data

All processing of calcium imaging data was carried out using custom software written in Python. To correct for movement in the *x-y* plane, the frames in each image sequence were registered to a reference image using phase-only correlation [S7]. Each of these reference images was also manually registered to a single stack taken before imaging in order to correct for slow drift occurring between movies. Regions of interest (ROIs) were selected for each neuron and the mean fluorescence within each ROI was calculated for every frame. The raw fluorescence traces from each ROI were then de-noised by Kalman smoothing, and the baseline fluorescence was estimated by finding the minimum fluorescence values within a 100 frame moving window. The normalized change in fluorescence (ΔF/F) was then calculated as $(F-F_0)/F_0$, where F is the Kalman-smoothed fluorescence trace for each ROI, and F_0 is the running baseline estimate.

Analysis of spatial receptive fields

Spatial receptive fields (RFs) were constructed by summing the ΔF/F recorded over the first 5 s following stimulus onset for dots presented in each grid location. Each spatial RF was fitted with a two-dimensional Gaussian function:

$$f(x,y)=A\exp\left(-\left(a(x-x_0)^2+2b(x-x_0)(y-y_0)+c(y-y_0)^2\right)\right)+B$$

Where:

$$a=\frac{\cos^2\Theta}{2\sigma_x^2}+\frac{\sin^2\Theta}{2\sigma_y^2}, b=\frac{-\sin 2\Theta}{4\sigma_x^2}+\frac{\sin 2\Theta}{4\sigma_y^2}, c=\frac{\sin^2\Theta}{2\sigma_x^2}+\frac{\cos^2\Theta}{2\sigma_y^2}$$

Parameters A and B correspond to the amplitude and baseline response, x_0 and y_0 correspond to the center coordinates of the RF, σ^2_x and σ^2_y correspond to the variance in the major and minor axes, and Θ corresponds to the rotation. The center coordinates of the fit were constrained to fall within the stimulus window. To determine whether a neuron showed statistically significant spatial selectivity, we generated 1,000 surrogate RFs by randomly shuffling the x and y coordinates associated with each response. If the R^2 value for the Gaussian fit to the true RF fell within the top 5th percentile of R^2 values obtained from the shuffled data, we considered the neuron to show significant spatial selectivity. Only significantly selective neurons were included for subsequent analysis.

Pairwise bootstrap test
In order to control for the effect of spatial clustering amongst sister neurons we compared each pair of sister neurons with a spatially matched set of non-sister pairs. To be included in the set of matched controls for a given sister pair, each pair of non-sister neurons had to be situated in the same combination of tectal layers as the sister pair and had to be the same distance apart from one another as the sister pair (to within a tolerance of ±10 μm; **Fig. 4D**). We expressed the Δcenter value of each pair of sister neurons relative to its corresponding set of matched controls as a percentile (**Fig. 4E**). Percentile values less than the median represented sister pairs that had more similar RF center positions than the average matched control pair. To assess whether sister neurons are statistically more similar, we compared the mean percentile value across all sister pairs with 100,000 randomly generated mean percentiles. These mean percentiles were obtained by sampling from the set of all possible percentiles for each sister pair. This enabled us to calculate a p-value ($P < 0.001$, **Fig. 4F**), corresponding to the probability of drawing a mean percentile less than that observed for the real sister pairs by chance.

Supplemental References

S1. Ohtsuki, G., Nishiyama, M., Yoshida, T., Murakami, T., Histed, M., Lois, C., and Ohki, K. (2012). Similarity of Visual Selectivity among Clonally Related Neurons in Visual Cortex. Neuron 75, 65–72.

S2. Nieuwkoop, P. D., and Faber, J. (1994). Normal table of Xenopus laevis (Daudin) (New York: Garland Publishing).

S3. Straznicky, K., and Gaze, R. M. (1972). The development of the tectum in Xenopus laevis: an autoradiographic study. J. Embryol. Exp. Morphol. *28*, 87–115.

S4. Richards, B. A., Voss, O. P., and Akerman, C. J. (2010). GABAergic circuits control stimulus-instructed receptive field development in the optic tectum. Nat. Neurosci. 13, 1098–1106.

S5. Lázár, G., and Székely, G. (1967). Golgi studies on the optic center of the frog. J. Hirnforsch. 9, 329–44.

S6. Longair, M. H., Baker, D. A., and Armstrong, J. D. (2011). Simple Neurite Tracer: open source software for reconstruction, visualization and analysis of neuronal processes. Bioinformatics *27*, 2453–4.

S7. Guizar-Sicairos, M., Thurman, S. T., and Fienup, J. R. (2008). Efficient subpixel image registration algorithms. Opt. Lett. 33, 156–8.
