# Supplementary material for: Clonal Relationships Impact Neuronal Tuning within a Phylogenetically Ancient Vertebrate Brain Structure
Source: Curr Biol. 2014 Aug 18;24(16):1929–33. doi: 10.1016/j.cub.2014.07.015 (PMC4151134; doi:10.1016/j.cub.2014.07.015)
Supplement: Document S2. Article plus Supplemental Information [file mmc2.pdf]

Current Biology 24, 1929–1933, August 18, 2014 ©2014 The Authors http://dx.doi.org/10.1016/j.cub.2014.07.015

Report

Clonal Relationships Impact Neuronal Tuning within a Phylogenetically Ancient Vertebrate Brain Structure

Alistair M. Muldal,[1] Timothy P. Lillicrap,[1] Blake A. Richards,[1,2] and Colin J. Akerman[1,*]
[1]Department of Pharmacology, University of Oxford, Oxford OX1 3QT, UK
[2]Department of Cell and Systems Biology, University of Toronto, Toronto, ON M5S 3G5, Canada

Summary

Understanding how neurons acquire specific response properties is a major goal in neuroscience. Recent studies in mouse neocortex have shown that "sister neurons" derived from the same cortical progenitor cell have a greater probability of forming synaptic connections with one another [1, 2] and are biased to respond to similar sensory stimuli [3, 4]. However, it is unknown whether such lineage-based rules contribute to functional circuit organization across different species and brain regions [5]. To address this question, we examined the influence of lineage on the response properties of neurons within the optic tectum, a visual brain area found in all vertebrates [6]. Tectal neurons possess well-defined spatial receptive fields (RFs) whose center positions are retinotopically organized [7]. If lineage relationships do not influence the functional properties of tectal neurons, one prediction is that the RF positions of sister neurons should be no more (or less) similar to one another than those of neighboring control neurons. To test this prediction, we developed a protocol to unambiguously identify the daughter neurons derived from single tectal progenitor cells in *Xenopus laevis* tadpoles. We combined this approach with in vivo two-photon calcium imaging in order to characterize the RF properties of tectal neurons. Our data reveal that the RF centers of sister neurons are significantly more similar than would be expected by chance. Ontogenetic relationships therefore influence the fine-scale topography of the retinotectal map, indicating that lineage relationships may represent a general and evolutionarily conserved principle that contributes to the organization of neural circuits.

Results and Discussion

To examine whether lineage-based rules contribute to functional circuit organization in the optic tectum, we developed a method for labeling a single neuronal clone per animal, which enabled us to definitively identify sister tectal neurons. All animal procedures were conducted in accordance with UK Home Office regulations. Individual tectal progenitor cells in the proliferative zone [8] of stage 44–47 *Xenopus laevis* tadpoles (7–16 days postfertilization) were targeted for single-cell electroporation with a dextran-conjugated red fluorescent dye (Figure 1A; Supplemental Experimental Procedures available online) [9]. This dye does not leak out of cells and is only passed on to daughter cells [10, 11]. To ensure that a single neuronal clone was labeled, we conducted in vivo two-photon imaging at different time points. The first imaging was conducted 1–3 hr after electroporation to be certain that only one progenitor had taken up the dextran (Figure 1B). From a total of 438 animals in which we confirmed that a single progenitor cell was labeled, 103 contained two or more labeled sister neurons when the animal was reimaged 6–19 days later at stage 49 or 50 (Figure 1C).

To reveal the architecture of the tectum and to probe the functional properties of tectal neurons, we then injected the calcium indicator dye Oregon Green BAPTA1-AM (OGB1-AM) into the region encompassing the dextran-labeled neurons. Images taken before and after the OGB1-AM injection enabled us to confirm 45 animals in which the dextran-labeled neurons could still be clearly distinguished, and the different tectal layers were clearly demarcated [12, 13] (Figure 2A). Each clone was comprised of 2–7 fluorescently labeled neurons. The majority of clones (25 out of 45; 56%) spanned multiple cell-dense layers of the tectum, and, in the remainder (20 out of 45; 44%), the neurons were restricted to the same layer (Figure 2B). Across all clones, there was a strong tendency for neurons derived from the same progenitor to be situated within nearby cell-dense layers ($p < 2 \times 10^{-5}$, bootstrap test; Figure 2C; Supplemental Experimental Procedures).

We then used two-photon calcium imaging to assess the response properties of clonally related neurons. We mapped spatial receptive fields (RFs) by simultaneously recording visually evoked calcium responses in both dextran-labeled and nonlabeled tectal neurons in the same animals (Figures 3A–3C; Supplemental Experimental Procedures) [14–16]. For clones to be included in the analysis, labeled neurons were required to exhibit robust spatially localized RFs, as determined statistically by fitting each RF with a 2D Gaussian function (Figures 3D and 3E; Supplemental Experimental Procedures). Clones in which only one neuron satisfied these criteria had to be excluded because sister comparisons were not possible. Under these criteria, we obtained a subset of animals with significant spatially selective responses in multiple dextran-labeled sister neurons and in a large fraction of nonlabeled neighboring neurons (11 labeled neurons, 531 nonlabeled neurons, four animals). Importantly, there was no significant difference between labeled and nonlabeled neurons in terms of their response amplitudes, the quality (R^2) of the RF fits, or the eccentricity of their RF centers (Figure 3F).

These data provided the opportunity to test whether clonal relationships influence the RF properties of tectal neurons. To quantify functional differences between pairs of tectal neurons, we computed the euclidean distance between the centers of their fitted RFs (Δcenter; Figure 4A). As expected, given the retinotopic organization of the tectum, there was a significant positive correlation between the spatial separation of pairs of neurons and their Δcenter values (Figure 4A). Although pairs of sister neurons had smaller Δcenter values than nonsister pairs (Figure 4B), they

*Correspondence: colin.akerman@pharm.ox.ac.uk
This is an open access article under the CC BY license (http://creativecommons.org/licenses/by/3.0/).

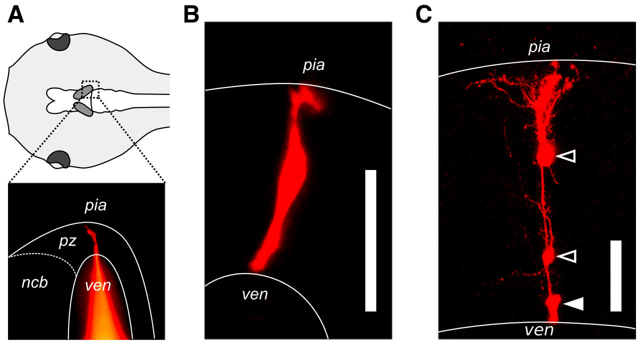

Figure 1. Lineage Tracing of Individual Tectal Progenitor Cells

(A) Schematic dorsal view of a tadpole's head (top) illustrating the positions of the two optic tecta (shaded); epifluorescence image (bottom) showing the electroporation of a single tectal progenitor cell with a fluorescently conjugated dextran (red). Region corresponds to dashed box above. ven, ventricle; pz, proliferative zone; ncb, neuronal cell bodies.

(B) Two-photon image showing a single tectal progenitor cell captured 2 hr postelectroporation. The scale bar represents 50 μm.

(C) Image of a tectal clone consisting of one radial progenitor cell (solid arrowhead) and two daughter neurons (open arrowheads), collected 10 days postelectroporation. The scale bar represents 50 μm.

also tended to be situated closer to one another within the tectum (Figure 4C). Thus, to assess the effect of clonal relationships, it was crucial for us to control for the bias introduced by this spatial clustering. We therefore compared each pair of sister neurons with a spatially matched set of nonsister control pairs (Figure 4D; Supplemental Experimental Procedures). The spatially matched control pairs had to be situated in the same combination of tectal layers, and they had to be the same distance apart as the corresponding sister pairs, to within a tolerance of ±10 μm. We expressed the degree of functional similarity between each sister pair relative to its matched controls as a percentile (Figure 4E; Supplemental Experimental Procedures). Percentile values less than the median indicate neuron pairs that had more similar RF center positions than their average matched control pair. Across the population, we found that sister pairs had a significantly smaller average percentile value than would be expected by chance ($p < 0.001$, bootstrap test; Figures 4F and S1A; Supplemental Experimental Procedures). Thus, pairs of sister neurons show more similar RF center positions than would be expected, given their spatial proximity within the tectum. This bias was also evident when we excluded pairs of neurons located within the same tectal layer (Figure S1B).

Our data demonstrate that sister neurons within the optic tectum have significantly more similar RF centers than nonsisters, indicating that neuronal lineage relationships influence the fine-scale topography of the retinotectal map. This is consistent with the observation that clonally related neurons can show similar orientation preferences in mouse visual cortex [3, 4]. The functional significance of such a mechanism is not yet fully understood, but it has been proposed that lineage relationships contribute to the establishment of precise canonical microcircuits [1, 5]. Given that retinotopic map formation has been shown to be controlled by molecular gradients

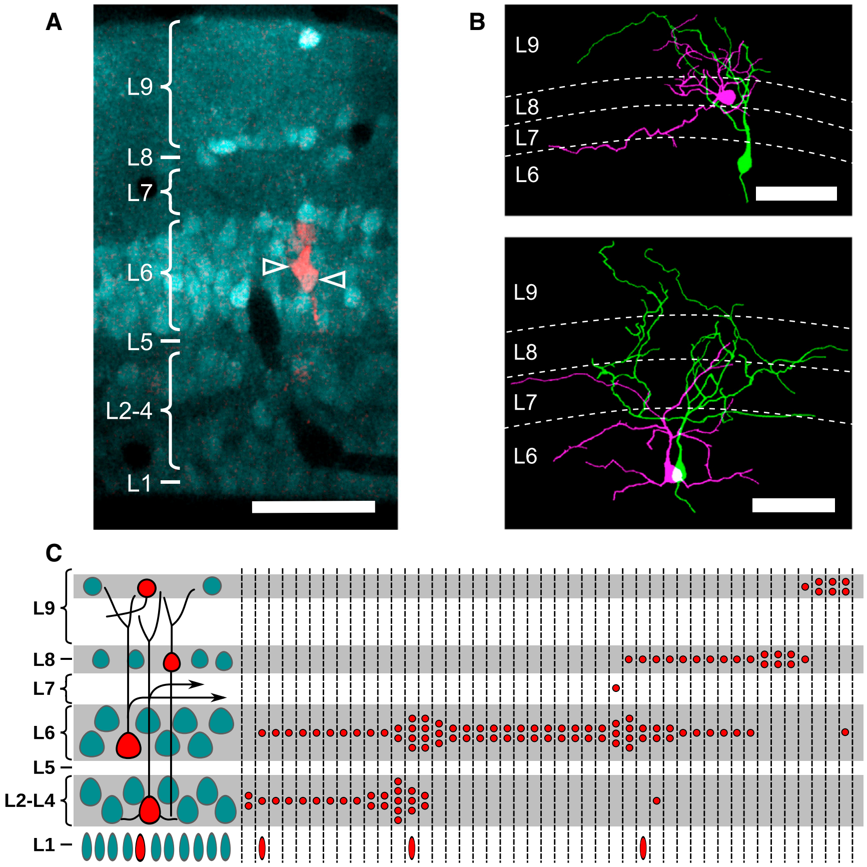

Figure 2. Morphology and Laminar Distribution of Tectal Sister Neurons

(A) Two-photon image showing a pair of labeled sister neurons (red, open arrowheads) within a tectum loaded with OGB1-AM (cyan). The scale bar represents 50 μm. Boundaries of the nine tectal layers are annotated on the left.

(B) Example morphological reconstructions of labeled sister neurons located in different tectal layers (top) and in the same tectal layer (bottom). Dotted white lines denote the positions of the layer boundaries, as determined from the OGB1-AM loading. Scale bars represent 50 μm.

(C) Diagram showing the main tectal layers and cell types (left) and laminar fates of labeled sister neurons (right; n = 45 clones). Cell-dense layers are gray; neuropil layers are white. Red circles within each dashed column represent layer positions of daughter neurons generated by a single progenitor cell.

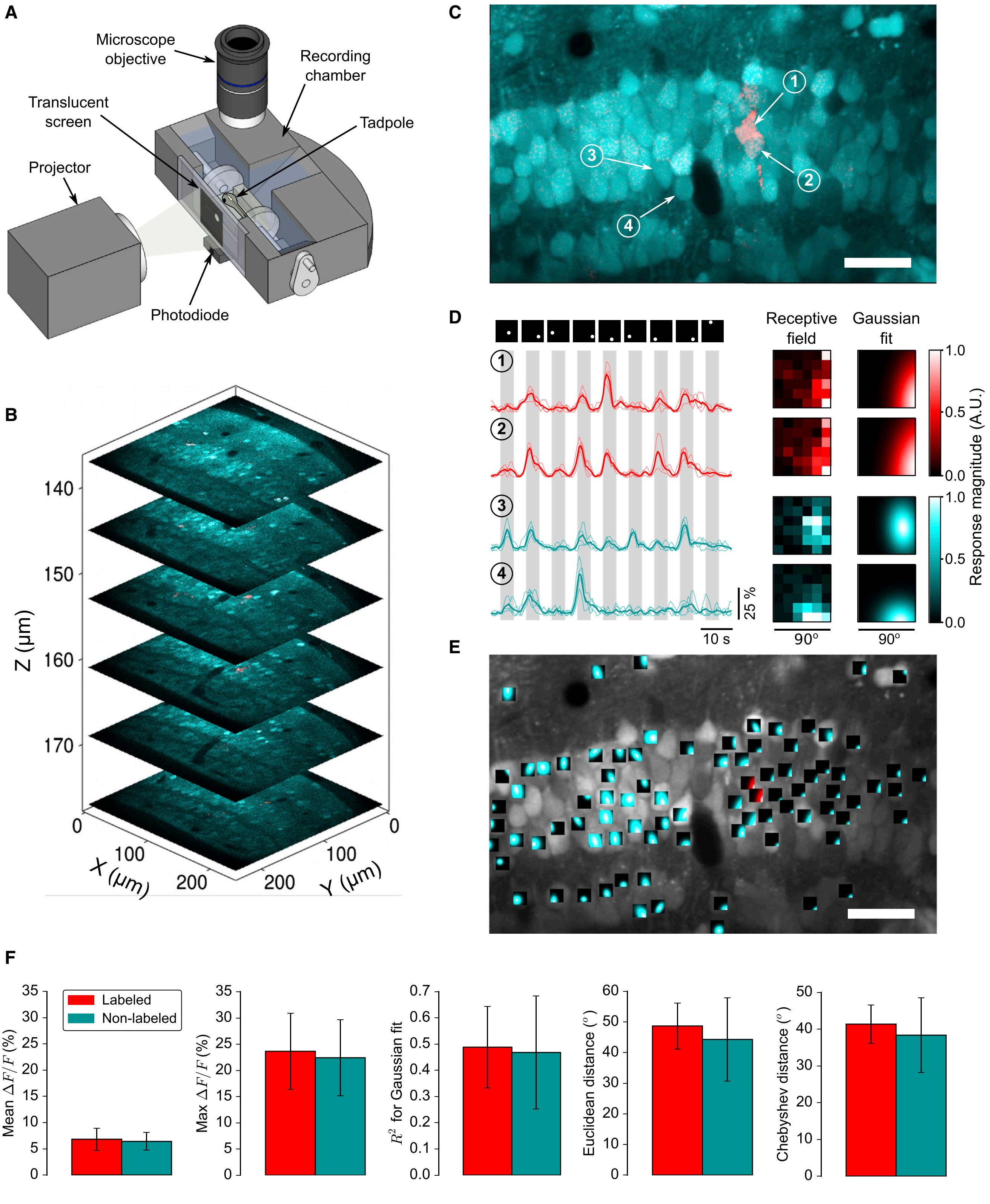

Figure 3. Two-Photon Calcium Imaging of Sister Neurons and Nearby Nonsister Neurons in the Optic Tectum

(A) Experimental setup for in vivo calcium imaging and visual stimulation.

(B) Two-photon stack through a region of tectum containing a single clone (red) and loaded with OGB1-AM (cyan). The z axis represents depth relative to the pial surface.

(C) Single plane containing two dextran-labeled sister neurons. The scale bar represents 50 μm.

(legend continued on next page)

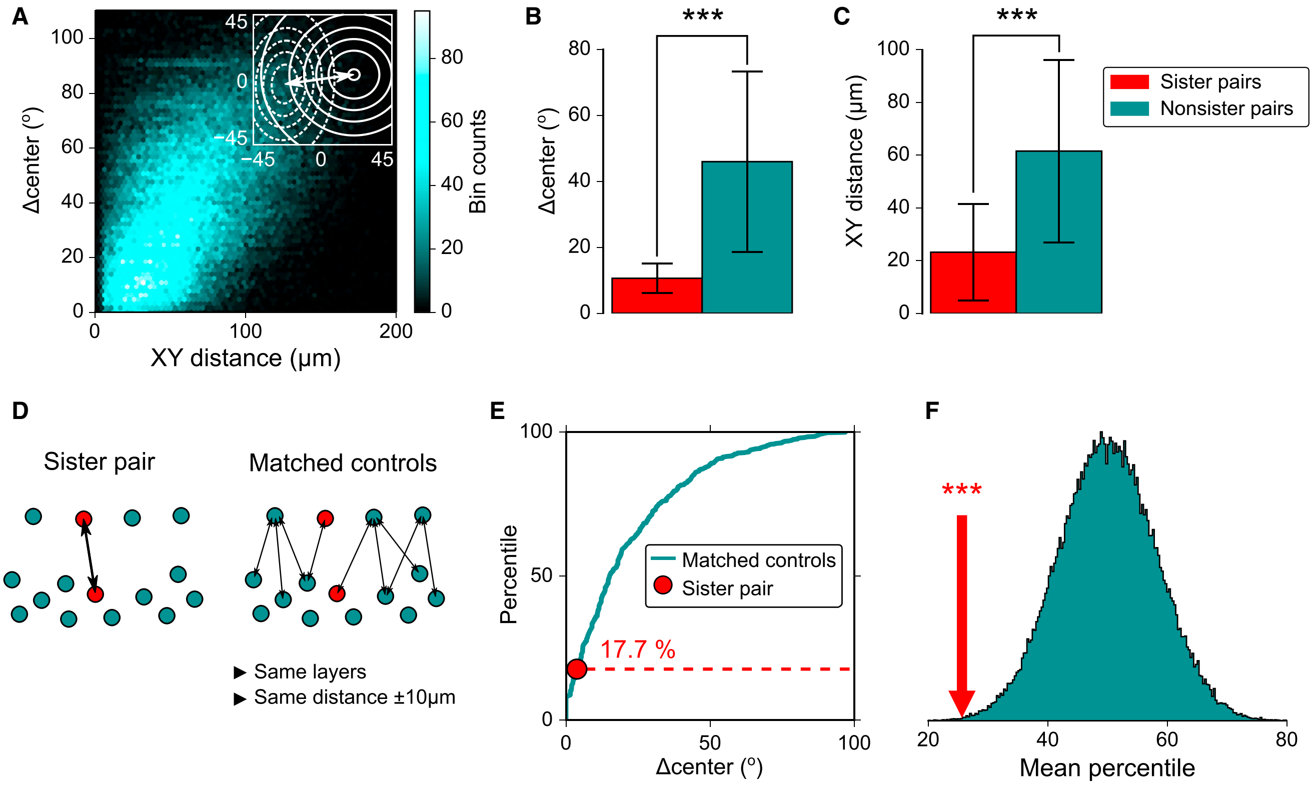

Figure 4. Sister Neurons in the Optic Tectum Have More Similar Spatial RFs Than Nonsisters

(A) Relationship between spatial distance and Δcenter value for all pairs of tectal neurons (***$p < 0.001$, $\rho = 0.45$, Spearman correlation). Inset illustrates how the Δcenter value was computed for a pair of RFs.

(B) Δcenter values for sister and nonsister pairs of tectal neurons (data indicate mean ± SD; n = 13 clonal pairs and n = 72,546 nonclonal pairs from four animals in which a single clone was labeled; ***$p < 0.001$, Mann-Whitney U test).

(C) Spatial distances between somata of sister and nonsister pairs (***$p < 0.001$, U test).

(D) Schematic showing a pair of sister neurons and its corresponding set of spatially matched nonsister control pairs.

(E) Cumulative distribution of Δcenter values for an example pair of sister neurons and its set of matched nonsister control pairs.

(F) Pairwise bootstrap test confirms that sister neurons have more similar RF center positions than would be expected, given their spatial proximity within the tectum. Cyan distribution represents a random sample of mean percentile values (Supplemental Experimental Procedures). The mean percentile for sister pairs (red arrow) was significantly smaller than would be expected by chance (***$p < 0.001$, bootstrap test).

and neuronal activity [17, 18], the influence of lineage upon a tectal neuron's functional properties could reflect the inheritance of a particular profile of gene expression [19, 20] and/or activity-dependent processes [2, 4]. Fundamentally, the fact that clonal relationships influence responses in the optic tectum, an ancient brain structure that is common to all vertebrates, indicates that lineage relationships may represent a general and evolutionarily conserved principle that contributes to the organization of neural circuits.

Supplemental Information

Supplemental Information includes Supplemental Experimental Procedures and one figure and can be found with this article online at http://dx.doi.org/10.1016/j.cub.2014.07.015.

Author Contributions

A.M.M., B.A.R., and C.J.A. designed the experiments. A.M.M. and B.A.R. performed the experiments. A.M.M. and T.P.L. performed the analysis. A.M.M. and C.J.A. wrote the paper.

Acknowledgments

This work was supported by a grant from the Biotechnology and Biological Sciences Research Council (BB/E0154761), and the research leading to these results has received funding from the European Research Council under the European Community's Seventh Framework Programme (FP7/2007-2013; ERC grant agreement number 243273). A.M.M. and B.A.R. were supported by Wellcome Trust Doctoral Fellowships, a Postgraduate Scholarship from the Natural Sciences and Engineering Research Council of Canada, and a Banting Postdoctoral Fellowship.

(D) Example traces showing visually evoked calcium responses recorded from the neurons labeled in (C). Thin lines denote single trials; thick lines denote the mean response across trials. The corresponding visual stimuli are shown above. Raw spatial RFs and Gaussian fits corresponding to these neurons are also shown (right).

(E) Fitted spatial RF maps obtained simultaneously from the two labeled sister neurons (red) and 83 nearby nonsister tectal neurons (cyan) shown in (C), superimposed onto their respective soma positions. The scale bar represents 50 μm.

(F) Population data showing that clonally labeled tectal neurons do not differ from nonlabeled neurons in terms of their response magnitude (mean ΔF/F, $p = 0.35$; maximum ΔF/F, $p = 0.31$; n = 11 labeled neurons and n = 531 nonlabeled neurons; Mann-Whitney U test), their spatial selectivity (R^2 values for Gaussian RF fits, $p = 0.34$), or the eccentricities of their RFs, as measured by either the euclidean ($p = 0.16$) or Chebyshev ($p = 0.24$) distance from the center of the stimulus area to the center of the fitted RF. Plots indicate mean ± SD.

Received: May 28, 2014
Revised: July 7, 2014
Accepted: July 7, 2014
Published: August 7, 2014

References

1. Yu, Y.-C., Bultje, R.S., Wang, X., and Shi, S.-H. (2009). Specific synapses develop preferentially among sister excitatory neurons in the neocortex. Nature *458*, 501–504.
2. Yu, Y.-C., He, S., Chen, S., Fu, Y., Brown, K.N., Yao, X.-H., Ma, J., Gao, K.P., Sosinsky, G.E., Huang, K., and Shi, S.H. (2012). Preferential electrical coupling regulates neocortical lineage-dependent microcircuit assembly. Nature *486*, 113–117.
3. Ohtsuki, G., Nishiyama, M., Yoshida, T., Murakami, T., Histed, M., Lois, C., and Ohki, K. (2012). Similarity of visual selectivity among clonally related neurons in visual cortex. Neuron *75*, 65–72.
4. Li, Y., Lu, H., Cheng, P.L., Ge, S., Xu, H., Shi, S.-H., and Dan, Y. (2012). Clonally related visual cortical neurons show similar stimulus feature selectivity. Nature *486*, 118–121.
5. Gao, P., Sultan, K.T., Zhang, X.-J., and Shi, S.-H. (2013). Lineage-dependent circuit assembly in the neocortex. Development *140*, 2645–2655.
6. Butler, A.B., and Hodos, W. (2005). Comparative Vertebrate Neuroanatomy, Second Edition (Hoboken: John Wiley & Sons).
7. Gaze, R.M. (1958). The representation of the retina on the optic lobe of the frog. Q. J. Exp. Physiol. Cogn. Med. Sci. *43*, 209–214.
8. Straznicky, K., and Gaze, R.M. (1972). The development of the tectum in Xenopus laevis: an autoradiographic study. J. Embryol. Exp. Morphol. *28*, 87–115.
9. Haas, K., Sin, W.C., Javaherian, A., Li, Z., and Cline, H.T. (2001). Single-cell electroporation for gene transfer in vivo. Neuron *29*, 583–591.
10. Wetts, R., and Fraser, S.E. (1988). Multipotent precursors can give rise to all major cell types of the frog retina. Science *239*, 1142–1145.
11. Bronner-Fraser, M., and Fraser, S.E. (1988). Cell lineage analysis reveals multipotency of some avian neural crest cells. Nature *335*, 161–164.
12. Lázár, G., and Székely, G. (1967). Golgi studies on the optic center of the frog. J. Hirnforsch. *9*, 329–344.
13. Székely, G., and Lázár, G. (1976). Cellular and synaptic architecture of the optic tectum. In Frog Neurobiology, R. Llinás and W. Precht, eds. (Heidelburg: Springer-Verlag), pp. 407–434.
14. Niell, C.M., and Smith, S.J. (2005). Functional imaging reveals rapid development of visual response properties in the zebrafish tectum. Neuron *45*, 941–951.
15. Richards, B.A., Voss, O.P., and Akerman, C.J. (2010). GABAergic circuits control stimulus-instructed receptive field development in the optic tectum. Nat. Neurosci. *13*, 1098–1106.
16. Dunfield, D., and Haas, K. (2010). In vivo single-cell excitability probing of neuronal ensembles in the intact and awake developing Xenopus brain. Nat. Protoc. *5*, 841–848.
17. Luo, L., and Flanagan, J.G. (2007). Development of continuous and discrete neural maps. Neuron *56*, 284–300.
18. Cang, J., and Feldheim, D.A. (2013). Developmental mechanisms of topographic map formation and alignment. Annu. Rev. Neurosci. *36*, 51–77.
19. Jeffries, A.R., Perfect, L.W., Ledderose, J., Schalkwyk, L.C., Bray, N.J., Mill, J., and Price, J. (2012). Stochastic choice of allelic expression in human neural stem cells. Stem Cells *30*, 1938–1947.
20. Coskun, V., Tsoa, R., and Sun, Y.E. (2012). Epigenetic regulation of stem cells differentiating along the neural lineage. Curr. Opin. Neurobiol. *22*, 762–767.

Current Biology, Volume *24*

Supplemental Information

Clonal Relationships Impact Neuronal Tuning within a Phylogenetically Ancient Vertebrate Brain Structure

Alistair M. Muldal, Timothy P. Lillicrap, Blake A. Richards, and Colin J. Akerman

Figure S1

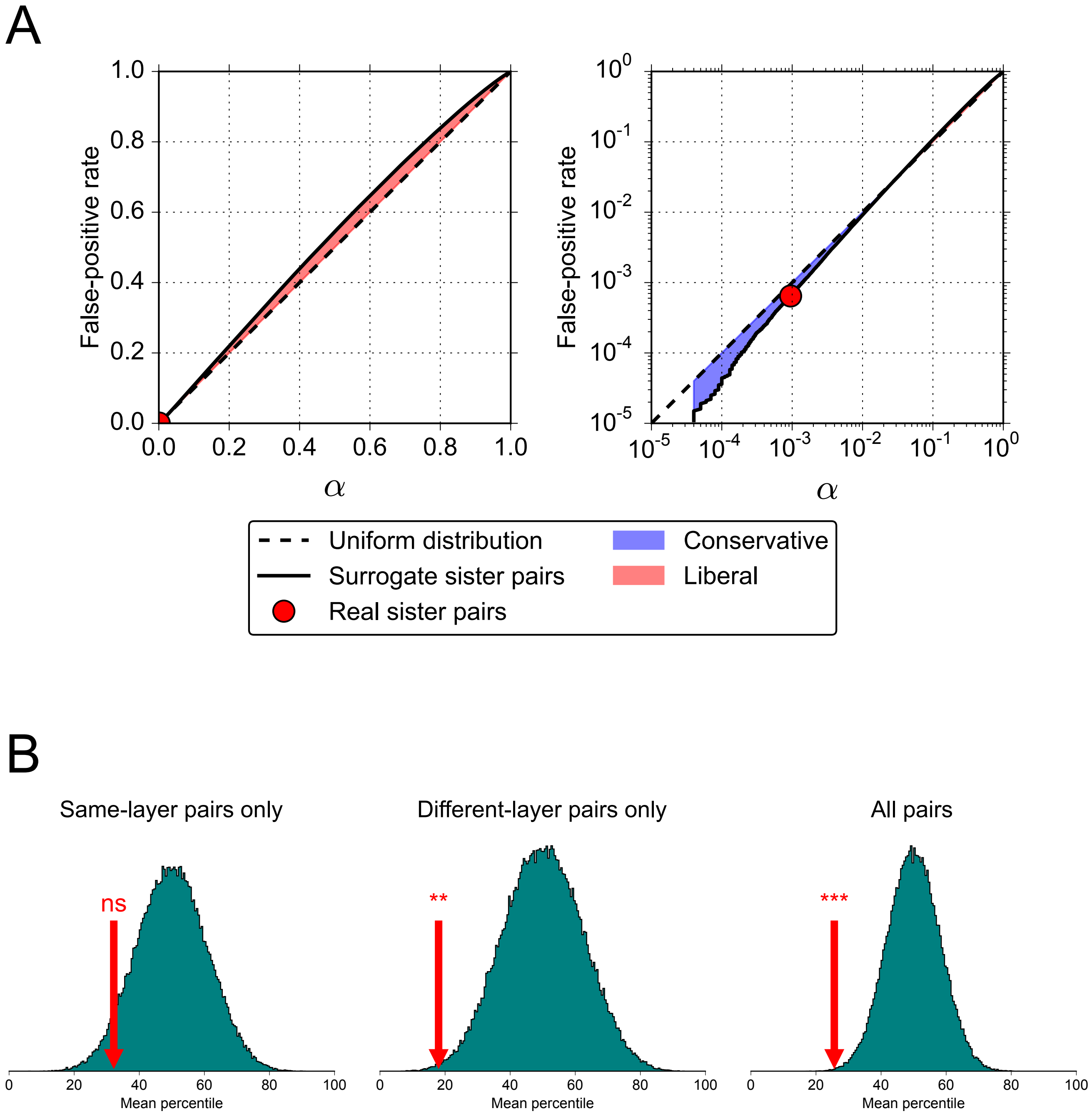

A
False-positive rate
α
Uniform distribution
Surrogate sister pairs
Real sister pairs
Conservative
Liberal
B
Same-layer pairs only
Different-layer pairs only
All pairs
ns
**
***
Mean percentile

Figure S1, related to Figure 4

(A) To confirm that our pairwise bootstrap test did not exhibit a bias towards Type I errors (i.e. incorrectly rejecting the null hypothesis that sister pairs do not show smaller Δcenter values than non-sister pairs), we performed an additional statistical control to estimate the false-positive rate of the test [S1]. For each real pair of sister cells we randomly selected one of its spatially matched non-sister control pairs to serve as a surrogate. We then treated each surrogate sister pair to the same analysis we performed on the real sister pairs. By doing this for 1 million sets of surrogate pairs we generated a sample of p-values from pairs of cells that were similar to real sister pairs in terms of their relative spatial positions, but that lacked any specifically defined lineage relationship. The plots show the cumulative probability distributions for the p-values we obtained from the surrogate pairs (solid black lines), plotted on both linear (left) and log-log (right) axes. These are equivalent to plotting the estimated false-positive rate against the critical value (α) of the pairwise bootstrap test. If the test were perfectly unbiased, the p-values we obtained from the surrogate pairs would be uniformly distributed between 0 and 1, such that the false-positive rate is exactly equal to the critical value of the test (dashed lines). For example, when $\alpha = 0.05$, the probability that a perfectly unbiased test would incorrectly reject the null hypothesis is exactly 5%. If our pairwise bootstrap test is performed with $\alpha = 0.05$, the corresponding false-positive rate of 0.052 means that there is a 5.2% chance of incorrectly rejecting the null hypothesis, and therefore the test is slightly too liberal at this critical value. However, we find that p-values less than 0.0286 are actually under-represented relative to a uniform distribution (blue shaded region). Since the p-value we obtained for the real sister pairs falls within this region ($P < 0.00095$, red circle), our original p-value was too conservative. We can correct for this bias by reading off the false-positive rate at $\alpha = 0.00095$. This gives a corrected p-value of 0.00064, allowing us to safely reject the null hypothesis at the 0.1% level.

(B) To examine whether the bias for clonally related neurons to have similar RF center positions could be detected for cells either within or across tectal layers, we divided our set of 13 sister pairs into two subsets: one consisting of pairs where both neurons were within the same layer, and the other consisting of pairs where the neurons were situated in different layers. We then performed a pairwise bootstrap test separately on each subset of pairs (Supplemental Experimental Procedures). The plots show the results of these bootstrap tests, plotted according to the conventions of Fig. 4F. The result obtained for all sister pairs is also shown for reference (identical to Fig. 4F). We found that different-layer pairs showed a strong and highly statistically significant bias towards having more similar RF center positions (mean percentile = 18.0%; $P < 0.005$; n = 6 pairs). Although same-layer pairs also had a lower average percentile than their matched controls, the magnitude of the effect was smaller and did not reach statistical significance at the 5% level (mean percentile = 32.2%; $P = 0.051$; n = 7 pairs).

Supplemental Experimental Procedures

Single-cell electroporation

All animal procedures were conducted in accordance with UK home office regulations. Wild-type *Xenopus laevis* tadpoles staged between 44 and 47 according to [S2] were anesthetized in 0.01 % wt/vol tricaine methane sulphonate (MS222). A glass micropipette filled with a fluorescently-labeled dextran (Alexa Fluor 594 dextran conjugate, 10,000 MW, anionic, fixable; Molecular Probes) was inserted into the right ventricle. The tip was then advanced until it touched the ventricular wall of the proliferative zone, located at the medial-posterior boundary of the tectum [S3], and a single positive voltage pulse (+1 V amplitude, 2 ms duration) was delivered using an Axoporator 800A (Axon Instruments). Within 3 h of electroporation animals were screened under a two-photon microscope and any that showed either multiple fluorescent cells, or cells that did not morphologically resemble radial progenitor cells, were discarded.

In vivo imaging

The right tectum of each tadpole was bulk-loaded with the calcium-sensitive fluorescent indicator Oregon Green 488 BAPTA-1 AM (OGB1-AM; Molecular Probes). This was prepared at a concentration of 10 mM in DMSO containing 20 % pluronic acid (Sigma), which was further diluted 10:1 in calcium-free Ringer's solution (in mM: 150 NaCl, 2.5 KCl, 10 HEPES). One microlitre of Alexa 594 red fluorescent dye (Molecular Probes) was added to aid visualization during loading. The tadpole was anesthetized in MS222, and the dye solution was pressure-injected into the tectal parenchyma through a glass micropipette using a Picospritzer III (General Valve). The tadpole was allowed to fully recover in normal rearing solution (1 – 3 hrs) and then was immersed in rearing solution containing 0.3% wt/vol pancuronium dibromide (Sigma) for 15 min to prevent muscle movements during functional imaging. The tadpole was positioned within a custom-made recording chamber with the eye contralateral to the loaded tectum facing a translucent screen onto which visual stimuli were projected (**Fig. 3A**). All imaging was carried out using a custom-built two-photon microscope consisting of a modified confocal scan unit (Olympus FV300) and a Ti:Sapphire laser (Newport Spectra-Physics Mai Tai HP). The laser wavelength was tuned to 810 nm to simultaneously excite both the Alexa 594-conjugated dextran and the OGB1-AM calcium dye. Emission from the red and green fluorophores was separated using a dichroic mirror (Q595LP, Chroma) and corresponding bandpass filters (HQ645/75M and HQ525/50M-2P, Chroma) and detected using separate photomultplier tubes. During visual stimulation, *x-y* raster scans were captured at 2 Hz over a rectangular region of tectum measuring approximately 170x110 μm, at depths of 140-260 μm from the pial surface.

Visual stimulation

The setup for visual stimulation was similar to that previously described [S4]. Visual stimuli were generated using custom software written in Python and were projected onto the window of the imaging chamber using an LCD projector (Samsung SP-P310ME; 800 x 600 px, 60 Hz refresh rate) masked by a Wratten Filter 32 (Kodak). The tadpole was positioned such that the stimulus window covered the central 90° x 90° of the tadpole's left visual field. To ensure that the imaging data was precisely time-locked to the presentation of the visual stimuli, a photodiode positioned in front of the projector was used to trigger the acquisition of each frame. The stimuli consisted of bright dots with a radius of 12.9^{o} which briefly appeared against a dark background. The minimum and maximum luminance values of the image projected onto the screen were 0.06 cd/m^2 and 1025 cd/m^2, respectively. During each stimulus epoch, a dot appeared at a pseudorandom location within a 6 x 6 grid for 2 s, followed by a 10 s blank period. Each stimulus position was repeated 3-5 times per

experiment.

Laminar and morphological analysis of sister neurons

Tectal layers were identified based on cell density and neuropil staining from two-photon stacks through OGB1-AM loaded tissue, in accordance with [S5]. We performed a bootstrap test to establish whether clonally-related cells tend to occupy neighboring layers of the tectum. Only layers 2-4, 6, 8 and 9, which are neuron-dense, were included in this analysis. Layers 2-4 cannot be reliably distinguished at this developmental stage and were therefore grouped together. From our experimental data set of 45 clones, we generated one million surrogate data sets in which we randomly shuffled the neurons within each layer across the clones. The real and surrogate data were then compared in terms of the average laminar distance between all possible pairs of neurons within each clone, where distance was defined as the absolute difference in the laminar positions of the neurons. We obtained a p-value by calculating the proportion of surrogate data sets that had a smaller average pairwise distance than the experimental data. In a subset of clones where the neuronal processes were sufficiently well-filled by the fluorescent dextran (n = 15/45), labeled sister neurons were reconstructed from two-photon stacks using the Simple Neurite Tracer plugin for FIJI [S6].

Processing of calcium imaging data

All processing of calcium imaging data was carried out using custom software written in Python. To correct for movement in the *x-y* plane, the frames in each image sequence were registered to a reference image using phase-only correlation [S7]. Each of these reference images was also manually registered to a single stack taken before imaging in order to correct for slow drift occurring between movies. Regions of interest (ROIs) were selected for each neuron and the mean fluorescence within each ROI was calculated for every frame. The raw fluorescence traces from each ROI were then de-noised by Kalman smoothing, and the baseline fluorescence was estimated by finding the minimum fluorescence values within a 100 frame moving window. The normalized change in fluorescence (ΔF/F) was then calculated as $(F-F_0)/F_0$, where F is the Kalman-smoothed fluorescence trace for each ROI, and F_0 is the running baseline estimate.

Analysis of spatial receptive fields

Spatial receptive fields (RFs) were constructed by summing the ΔF/F recorded over the first 5 s following stimulus onset for dots presented in each grid location. Each spatial RF was fitted with a two-dimensional Gaussian function:

$$f(x,y)=A\exp\left(-\left(a\left(x-x_0\right)^2+2\mathrm{b}\left(x-x_0\right)\left(y-y_0\right)+c\left(y-y_0\right)^2\right)\right)+B$$

Where:

$$a=\frac{\cos^2\Theta}{2\sigma_x^2}+\frac{\sin^2\Theta}{2\sigma_y^2},b=\frac{-\sin 2\Theta}{4\sigma_x^2}+\frac{\sin 2\Theta}{4\sigma_y^2},c=\frac{\sin^2\Theta}{2\sigma_x^2}+\frac{\cos^2\Theta}{2\sigma_y^2}$$

Parameters A and B correspond to the amplitude and baseline response, x_0 and y_0 correspond to the center coordinates of the RF, σ^2_x and σ^2_y correspond to the variance in the major and minor axes, and Θ corresponds to the rotation. The center coordinates of the fit were constrained to fall within the stimulus window. To determine whether a neuron showed statistically significant spatial selectivity, we generated 1,000 surrogate RFs by randomly shuffling the x and y coordinates associated with each response. If the R^2 value for the Gaussian fit to the true RF fell within the top 5th percentile of R^2 values obtained from the shuffled data, we considered the neuron to show significant spatial selectivity. Only significantly selective neurons were included for subsequent analysis.

Pairwise bootstrap test

In order to control for the effect of spatial clustering amongst sister neurons we compared each pair of sister neurons with a spatially matched set of non-sister pairs. To be included in the set of matched controls for a given sister pair, each pair of non-sister neurons had to be situated in the same combination of tectal layers as the sister pair and had to be the same distance apart from one another as the sister pair (to within a tolerance of ±10 μm; **Fig. 4D**). We expressed the Δcenter value of each pair of sister neurons relative to its corresponding set of matched controls as a percentile (**Fig. 4E**). Percentile values less than the median represented sister pairs that had more similar RF center positions than the average matched control pair. To assess whether sister neurons are statistically more similar, we compared the mean percentile value across all sister pairs with 100,000 randomly generated mean percentiles. These mean percentiles were obtained by sampling from the set of all possible percentiles for each sister pair. This enabled us to calculate a p-value ($P < 0.001$, **Fig. 4F**), corresponding to the probability of drawing a mean percentile less than that observed for the real sister pairs by chance.

Supplemental References

S1. Ohtsuki, G., Nishiyama, M., Yoshida, T., Murakami, T., Histed, M., Lois, C., and Ohki, K. (2012). Similarity of Visual Selectivity among Clonally Related Neurons in Visual Cortex. Neuron 75, 65–72.

S2. Nieuwkoop, P. D., and Faber, J. (1994). Normal table of Xenopus laevis (Daudin) (New York: Garland Publishing).

S3. Straznicky, K., and Gaze, R. M. (1972). The development of the tectum in Xenopus laevis: an autoradiographic study. J. Embryol. Exp. Morphol. *28*, 87–115.

S4. Richards, B. A., Voss, O. P., and Akerman, C. J. (2010). GABAergic circuits control stimulus-instructed receptive field development in the optic tectum. Nat. Neurosci. 13, 1098–1106.

S5. Lázár, G., and Székely, G. (1967). Golgi studies on the optic center of the frog. J. Hirnforsch. 9, 329–44.

S6. Longair, M. H., Baker, D. A., and Armstrong, J. D. (2011). Simple Neurite Tracer: open source software for reconstruction, visualization and analysis of neuronal processes. Bioinformatics *27*, 2453–4.

S7. Guizar-Sicairos, M., Thurman, S. T., and Fienup, J. R. (2008). Efficient subpixel image registration algorithms. Opt. Lett. 33, 156–8.
